# Postoperative blood transfusion is an independent predictor of acute kidney injury in cardiac surgery patients

**DOI:** 10.12860/jnp.2015.23

**Published:** 2015-10-01

**Authors:** Kristofer Freeland, Alireza Hamidian Jahromi, Lucas Maier Duvall, Mary Catherine Mancini

**Affiliations:** ^1^Department of Surgery, Louisiana State University Health Sciences Center, Shreveport, LA, USA

**Keywords:** Complication, Cardiac surgery, Acute kidney injury, Transfusion, Coronary artery bypass graft

## Abstract

*Background:* Acute kidney injury (AKI) is a serious complication of cardiac surgery with cardiopulmonary bypass (CPB) which increases postoperative morbidity and mortality.

*Objectives:* The study was designed to assess the incidence of AKI and associated risk factors in patients undergoing CPB ancillary to coronary artery bypass grafting (CABG), valve surgery, and combined CABG and valve surgery.

*Patients and Methods:* This Intuitional Review Board (IRB) approved retrospective study included patients with normal preoperative kidney function (Serum creatinine [sCr] <2.0 mg/dl) who underwent cardiac surgery with CPB between 2012 and 2014. Patients were divided into 2 groups: group I: Patients with cardiac surgery associated AKI (CS-AKI) (postoperative sCr >2 mg/dl with a minimal doubling of baseline sCr) and group II: Patients with a normal postoperative kidney function. Demographic data, body mass index (BMI), co-morbidities, hematologic/biochemical profiles, preoperative ejection fraction (%EF), blood transfusion history, and operative data were compared between the groups. Mean arterial pressure (MAP) was recorded during the operation and in the postoperative period. Δ-MAP was defined as the difference between pre-CPB-MAP and the CPB-MAP.

*Results:* 241 patients matched the inclusion criteria (CS-AKI incidence = 8.29%). Age, gender, BMI, %EF, and co-morbidities were not predictors of CS-AKI (*P* > 0.05). High preoperative sCr (*P* = 0.047), type of procedure (*P* = 0.04), clamp time (*P* = 0.003), pump time (*P* = 0.005) and history of blood transfusion within 14 days postsurgery (*P* = 0.0004) were associated with risk of CS-AKI. Pre-CPB-MAP, CPB-MAP, Δ-MAP, and ICU-MAP were not significantly different between the 2 groups. Male gender (OR: 5.53; *P* = 0.048), age>60 (OR: 4.54; *P* = 0.027) and blood transfusion after surgery (OR: 5.25; *P* = 0.0054) were independent predictors for postoperative AKI.

*Conclusions:* Age, gender and blood transfusion were independent predictors of cardiac surgery associated AKI.

**Implication for health policy/practice/research/medical education:**

In the current study, we assessed the incidence of acute kidney injury (AKI) and associated risk factors in patients undergoing cardiopulmonary bypass (CBP) ancillary to coronary artery bypass grafting (CABG), valve surgery, and combined CABG and valve surgery. The outcome of the research has implications in clinical practice especially in the intraoperative and postoperative care of the cardiac surgery patients.

## 1. Background

Cardiac surgery associated acute renal injury (CS-AKI) is a serious complication following cardiac surgeries including cardiopulmonary bypass (CPB). It has been shown to occur in up to 30% of patients, depending on the definition used, with 1%-2% of these patients requiring renal replacement therapy (RRT) (1). Even a small postoperative increase in serum creatinine (sCr) has been shown to increase morbidity and mortality following cardiac surgeries (1). CS-AKI requiring RRT has been shown to increase postoperative mortality to as high as 50% (1). Multiple factors have been implicated as contributors to postoperative AKI including advanced age (2,3), female gender (3,4) chronic kidney disease (5,7), time delay between heart catheterization and surgery (5,8), aortic cross clamp time (clamp time) (2), duration of CPB (pump time) (2,9,10), differences in the preoperative and intraoperative mean arterial pressure (MAP) (11), and blood transfusion following surgery (12-15). AKI is defined as a sCr greater than 2.0 with a minimum doubling of baseline sCr or a new requirement for dialysis.

## 2. Objectives

This study was designed to investigate the incidence of AKI after cardiac surgery with CPB, including coronary artery bypass grafting (CABG), aortic and mitral valve surgery, and combined CABG and valve surgery and associated risk factors in a cohort of patients without a history of chronic kidney disease.

## 3. Patients and Methods

In a single center, IRB approved, retrospective study, we evaluated patients with normal preoperative kidney function (sCr < 2.0 mg/dl) undergoing CPB ancillary to CABG, aortic or mitral valve surgery, or combined CABG and valve surgery in our center between January 1, 2012 and January 1, 2014. Four surgeons performed all cases over the 2-year period. All patients underwent continuous flow CPB using the Century Heart Lung Machine (Medtronics Inc., Minneapolis, MN). Priming volume consisted of 1500 mL Plasmlyte, 250 mL albumin, 10 000 units of heparin, 25 g mannitol, antibiotic (zinacef or vancomycin), and a protamine cocktail (50 mg Benadryl, 20 mg Pepcid, and 125 mg solumedrol). Flow rates were adjusted to achieve a target cardiac index of 2.4-2.6 L/min/m^2^ or a Systemic venous oxygen saturation >70%. Core body temperature was maintained above 30°C. Cardioplegia was performed with ExactaMix Cardioplegia solution (Baxa Corp., Englewood, CO).

The eligible patients were categorized into 2 groups based on serial sCr measurements for up to 14 days postoperation. The baseline sCr was defined as the last recorded sCr prior to surgery. Group I represents patients that developed CS-AKI and group II represents patients with normal postoperative renal function. Demographic data, body mass index (BMI) (kg/m^2^), co-morbidities, preoperative and postoperative hematologic and biochemical profiles, preoperative ejection fraction (%EF), incidence of blood transfusion during the 14 postoperative days, pre-transfusion hemoglobin, and time delay between heart catheterization and cardiac surgery were collected and compared between the groups.

Operative data was compared including the type of procedure performed (CABG, valves, or combined CABG and valve), operative time, CPB time (pump time), and aortic cross clamp time (clamp time) (all in minutes). The MAP was recorded every 15 minutes at different phases of the perioperative period (prior to CPB, during CPB, and in the postoperative period in the surgical intensive care unit (ICU)) and compared between the 2 groups. The pre-CPB-MAP was measured from the time the arterial line was placed until 15 minutes before initiation of CPB. The CPB-MAP was recorded during the time the patient was on bypass. ∆-MAP was defined as the difference between pre-CPB-MAP and the CPB-MAP. The ICU-MAP was recorded every 15 minutes for four hours starting with the first ICU-MAP recorded. The mean pre-CPB-MAP, CPB-MAP, ∆-MAP, and ICU-MAP were compared between the groups.

### 3.1. Ethical issues

The study protocol was in accordance with the Declaration of Helsinki and was approved by the Ethics Committee of Louisiana States University (LSU) Health Shreveport, United States.

### 3.2. Statistical analysis

Statistical analysis was performed using SPSS version 15 (SPSS Inc., Chicago, IL) statistical software package. Descriptive statistics were used to summarize the data. Fisher exact test, or chi-square test, and analysis of variance (ANOVA) were used for comparison of categorical variables when appropriate and student t test for the comparison of continuous variables. In a logistic regression model, variables were assessed to determine independent risk factors predicting CS-AKI in the patients. A P value <0.05 was considered significant.

## 4. Results

During this period 286 adult patients underwent cardiac surgery (CABG, aortic or mitral valve surgery, or combined CABG and valve surgery) at our center. Of these, 241 patients matched our inclusion criteria (male = 168, 69.7%) with an average age of 59.02 ± 10.3 years (range 26-86). Forty-five (n = 45) patients were excluded secondary to a history of chronic kidney disease (sCr>2.0 or on RRT) (n = 23), procedures other than CABG or valves (n=13), postoperative mortality within the first 24 hours (n = 5), or patients undergoing intraoperative circulatory arrest (n = 4). The incidence of AKI in our patients was 8.29% (20/241). Mean BMI in the patients was 30.25 ± 7.58 kg/m^2^(range 16.6-56.89). Patients’ characteristics (demographic data, BMI and co-morbidities) are compared between the 2 groups in Table 1. During the study period 186 (77.2%) CABG, 40 (16.6%) valve surgery and 15 (6.2%) combined CABG and valve surgeries were performed for the patients and were included in the study. The mean operative time, pump time and clamp time were 257.33 ± 57.99 (range 155-518), 115.97 ± 39.28 (range 34-260) and 73.76 ± 32.10 (range 18-218) minutes, respectively.

**Table 1:** Comparison of patient’s characteristics between the 2 groups of patients with cardiac surgery associated AKI (CS-AKI) (group I) and patients with a normal postoperative kidney function (group II)

**Patient’s Characteristics**	**Group I** **CS-AKI (n = 20)**	**Group II** **Non-AKI (n = 221)**	***P *** **value**
Demographics			
Gender (male)	17 (85%)	151 (68%)	0.13
Age (years)^a^	61.8 ± 10.7 (26-74)	58.7 ± 10.2 (24-86)	0.23
BMI (Kg/m^2^)^a^	31.2±8.57 (20-54.35)	30.16±7.5 (16-56.89)	0.59
Co-morbidities			
Diabetic	9 (45%)	88 (40%)	0.64
CHF	4 (20%)	52 (24%)	1.00
HTN	18 (90%)	185 (84%)	0.74
CAD	15 (75%)	189 (85%)	0.20
History of MI	4 (20%)	86 (39%)	0.14
History of CVA	4 (20%)	19 (8.6%)	0.10
History of A-fib	2 (10%)	10 (4.5%)	0.26
COPD	1 (5%)	35 (16%)	0.32

Abbreviations: CHF, congestive heart failure; HTN, hypertension; CAD, coronary artery disease; MI, myocardial infarction; CVA, cerebrovascular accident; A-fib, atrial fibrillation; BMI, body mass index; COPD, chronic obstructive pulmonary disease. ^a^ Figures are presented as mean ± standard deviation (range)

Age, gender, BMI, %EF, and co-morbid conditions were not predictors of AKI in our patients (*P* > 0.05 for all). The incidence of surgery within 48 hours of heart catheterization was also not different between the 2 groups. A higher preoperative baseline sCr, the type of procedure, pump time, clamp time, and the incidence of blood transfusion within 14 postoperative days were all associated with the development of CS-AKI in our patients. Our data did not show any difference in pre-CPB-MAP, CPB-MAP, ∆-MAP, or ICU-MAP between the two groups (Table 2).

**Table 2:** Univariate analysis comparing patient’s baseline characteristics, procedure types, intraoperative and perioperative variables and between the 2 groups of patients with cardiac surgery associated AKI (CS-AKI) (group-I) and patients with a normal postoperative kidney function (group II)

**Characteristics**	**Group I** **CS-AKI (n = 20)**	**Group II** **Non-AKI (n = 221)**	***P *** ** value**
Baseline characteristics			
%EF^a^	51.2±15.4 (15-75)	49.8±14.8 (10-80)	0.69
Baseline Cr^a^	1.17±0.27 (0.7-1.7)	1.03±0.25 (0.5-2)	0.047
Pre-op Hgb^a^	8.04±1.69 (6.2-12.6)	7.88±1.27 (5.2-11.5)	0.68
Pre-op Hct^a^	24.2±5.23 (18.1-38.4)	23.3±3.75 (15.3-35.4)	0.46
Pre-transfusion Hgb^a^	8.69±0.90 (7.5-10.8)	8.28±0.63 (6.7-9.7)	0.09
Surgery (≤48 h)	6 (30%)	38 (17%)	0.22
Blood transfusion (14 d)	12 (60%)	47 (21%)	0.0004
Procedure types			
CABG	11 (55%)	175 (79%)	0.04
Valves	6 (30%)	34 (15%)	
CABG + valves	3 (15%)	12 (5%)	
Intraoperative variables			
Operative time^a^	273.4±63.19 (186-423)	255.9± 56.9 (155-518)	0.24
Pump time^a^	149.6±48.1 (82-260)	112.8±36.9 (34-252)	0.003
Clamp time^a^	100.9±40.6 (40-218)	71.3±30.1 (18-188)	0.005
Perioperative MAP			
Pre-CPB-MAP^a^	77.8±9.39 (60.7-98)	78.8 (51-105.4) ± 8.51	0.66
CPB-MAP^a^	57.02±4.52 (46-64.6)	58.03±5.00 (45-81.2)	0.35
∆-MAP^a^	20.79± 10.57 (5.3-43)	19.94±12.36 (47-65)	0.73
ICU-MAP^a^	81.61±8.76 (70.3-102.8)	82.92±10.52 (61.4-118.2)	0.53

Abbreviations: %EF, cardiac ejection fraction; Cr, Creatinine; Pre-op Hgb, pre-operative hemoglobin; Pre-op Hct, pre-operative hematocrit; Surgery (≤48 h), surgery less than 48 hours from heart catheterization; Blood transfusion (14 d), blood transfusion within 14 postoperative days; CABG, coronary artery bypass grafting; Valves, valve surgery; CABG + valves, combined CABG and valve surgery; pump time, duration of CBP; clamp time, aortic cross clamp time; CPB, cardiopulmonary bypass; MAP, mean arterial pressure; ΔMAP, Difference between pre-CPB MAP and CPB MAP; ICU, intensive care unit. ^a^ Figures are presented as mean ± standard deviation (range).

While there was no difference in the pre-transfusion Hgb level between the groups, patients with CS-AKI had significantly higher number of cases who received blood transfusion (60%) compared with the patients without AKI (21%), (*P *= 0.0004). Multivariate logistic regression showed that male gender and Age >60 years were independent predictors for the development of CS-AKI and that blood transfusion within 14 postoperative days had an independent association with the development of CS-AKI (Table 3).

**Table 3:** Modeling of comparing patient’s demographics, co-morbidities, baseline characteristics, and the procedure types on the risk of cardiac surgery associated AKI (CS-AKI) in the patients

**Patients Characteristics**	**OR**	**95% CI**	***P *** va**lue**
Demographics			
Male gender	5.53	1.01-30.29	0.048
Age >60 years	4.54	0.059-0.85	0.027
BMI <30 kg/m^2^	0.62	0.18-2.05	0.43
Co-morbidities			
Diabetes	1.05	0.29-3.81	0.938
CHF	0.69	0.15-3.05	0.62
HTN	1.91	0.29-12.57	0.5
CAD	0.51	0.06-4.42	0.54
History of MI	1.75	0.47-6.47	0.4
COPD	0.16	0.02-1.49	0.11
Baseline characteristics			
Blood trans (14 d)	5.25	1.63-16.85	0.005
Base Cr ≥1.3	0.36	0.097-1.37	0.135
Operation types			
CABG	0.184	0.031-1.08	0.037
Valves	0.77	0.06-9.39	0.587
Surgery (≤48 h)	1.36	0.33-5.58	0.67

Abbreviations: OR, odds ratio; BMI, body mass index; CHF, congestive heart failure; HTN, hypertension; CAD, coronary artery disease; MI, myocardial infarction; COPD, chronic obstructive pulmonary disease; Blood Trans (14 d), Blood transfusion within 14 postoperative days; Cr, creatinine; CABG, coronary artery bypass grafting; Valves, valve surgery; Surgery (≤48 h), surgery less than 48 hours from heart catheterization.

## 5. Discussion

Cardiac surgery associated AKI is a serious complication of cardiac surgery with CBP that has been shown to increase morbidity and mortality (1). We studied the incidence and risk factors associated with the development of CS-AKI in patients with normal preoperative kidney function. We found no association between patient’s gender, age, BMI, or co-morbidities and the development of CS-AKI with univariate analysis. Our findings differ from some other studies that have shown female gender, reduced left ventricular function, congestive heart failure, diabetes, and chronic obstructive pulmonary disease (COPD) as risk factors for developing CS-AKI (2). Comparison of preoperative and postoperative patient’s variables also did not show any association between %EF, preoperative Hgb/Hct, or the incidence of surgery within 48 hours of heart catheterization and the risk of CS-AKI. There has been evidence that suggests that a lower preoperative hemoglobin level is independently associated with CS-AKI. Karkouti et al (14) reported that one-third of anemic patients (hemoglobin 10-12 g/dl) undergoing CBP in their study developed CS-AKI. The preoperative hemoglobin was 8.04 ± 1.69 in the CS-AKI group in our patients but did not have an association with AKI. Ranucci et al (8), found that angiography on the same day as cardiac surgery with CPB was an independent risk factor for developing CS-AKI. In our study we extended the time from angiography to surgery to 48 hours and found no association with CS-AKI. When comparing the MAP, our study did not show any difference in pre-CPB-MAP, CPB-MAP, ∆-MAP, or ICU-MAP between the 2 groups. The previous studies have been inconsistent in this regard. While Kanji and colleagues (11) found a ∆-MAP≥26 mm Hg to be independently associated with AKI, Haase et al (12) found no association between intra-operative MAP and CS-AKI. Azau et al (16) also reported that increasing the intraoperative MAP between 75-80 mm Hg did not change the incidence of CS-AKI in their patients. This is an area that currently has inconsistent findings and needs further evaluation. The mean ∆-MAP in the CS-AKI group of our study was 20.79 mm Hg, and it did not reach the 26 mm Hg as mentioned by Kanji et al (11), which may explain why we found no association between the MAP with CS-AKI. We also recorded the pre-CPB MAP on the day of surgery, starting with placement of the arterial line, instead of using the preoperative MAP from the outpatient setting that Kanji et al utilized in their study.

Our study showed that a higher baseline sCr was associated with CS-AKI, although we only looked at patients with a preoperative sCr < 2.0. This is consistent with the previous studies that have shown that a history of chronic kidney disease (1), lower estimated glomerular filtration rate (eGFR) (6), or a higher baseline sCr (7,9) are associated with an increased risk of developing CS-AKI. It has also been shown that a preoperative SCr ≥ 1.3 is an independent risk factor for CS-AKI and increases the risk of postoperative dialysis, morbidity, and mortality (7). Multivariate analysis of preoperative sCr ≥ 1.3 did not show an independent association with the development of CS-AKI in our study, but this could be due to the small number of patients that developed CS-AKI. In the current study we did not look at long-term outcomes.

Our study also demonstrated an association between the type of procedure and the development of CS-AKI. It has been shown in some previous studies that CABG only procedures have the lowest incidence (2%-5%) and that valvular surgery or a combined procedure has a much higher incidence (up to about 30%) of CS-AKI development (9). Although our study showed an association between the type of surgery and the development of CS-AKI in univariate analysis, multivariate Cox regression analysis did not confirm this association as an independent association. This is most likely due to the low number of combined procedures in the two groups of patients.

Longer pump times are a well-established risk factor for the development of CS-AKI, and it is thought to be due to prolonged hemodynamic variations in renal perfusion, reperfusion injury, and a pro-inflammatory state (2,9,10,13). Our study, similarly, found that longer pump times were a risk factor for developing CS-AKI. The association of longer aortic cross clamp times and the development of CS-AKI is inconsistent, but it has been shown in several studies (2). Embolic phenomena are thought to contribute to the development of AKI when found. Our study found that longer clamp times are a risk factor for developing CS-AKI.

Multiple logistic regression found age>60 years old and male gender to be independent risk factors and blood transfusion within 14 postoperative days to be independently associated with the development of CS-AKI in our patients. Multiple studies have found female gender to be associated with an increased risk of renal injury after cardiac surgery though the findings have been inconsistent (3), and several theories have attempted to explain the reason for this association. These have included advanced age of female patients when compared to males at the time of operation, a higher incidence of co-morbidities in female patients, or a higher pre-operative baseline sCr. Univariate analysis in our study found no association between gender and the development of postoperative CS-AKI; however, multivariate logistic regression did find that male gender was an independent risk factor for the development of CS-AKI. Mitter et al (4) found that female gender was associated with CS-AKI when using sCr as a marker for AKI, but once the baseline preoperative estimated glomerular filtration rate (eGFR) was included in multivariate modeling, the independent association was no longer present. We did not use eGFR in our study.

Most studies that have evaluated the relationship between the incidence of blood transfusions and the development of CS-AKI have shown an independent association (15). During storage, packed red blood cells (RBCs) undergo depletion of ATP and 2,3-diphosphoglycerate, accumulate pro-inflammatory molecules, free iron and hemoglobin, lose their ability to generate nitric oxide, and become less deformable (13). These changes, combined with the inflammatory state that occurs with transfusion, contribute to CS-AKI.

Our study showed that blood transfusion within the first 14 postoperative days was an independent risk factor for the development of CS-AKI. We excluded perioperative transfusions within the 24 hours of surgery, only looking at the incidence of postoperative transfusion. We also looked at the pre-transfusion Hgb level and found that, while there was no difference in the pre-transfusion Hgb level between the groups, patients with CS-AKI had a significantly higher incidence of blood transfusion (60%) compared with the patients without AKI (21%). This would indicate that some other clinical factors may have contributed to the decision to transfuse RBCs. Whatever has been the basis for the decision to transfuse some of the patients at a lower threshold, it seems that RBC transfusion has been associated with a significantly higher incidence of CS-AKI in our patients. Knowing other possible risks and potential complications associated with RBC transfusion, we suggest further evaluation of the pros and cons of RBC transfusion including the risk of CS-AKI in individual cardiac surgery patients before making the ultimate decision for transfusion in such cases. Our study does not show whether RBC transfusion has a causal relationship as well as being a risk factor for CS-AKI in our patients. Further studies should be performed to clarify such relationship.

## 6. Conclusions

A higher baseline sCr, type of surgery, postoperative blood transfusion, clamp time and pump time were risk factors for CS-AKI in our patient population. Age > 60 years and male gender were independent predictors of CS-AKI in our patients, and a blood transfusion within 14 postoperative days was found to have an independent association with CS-AKI.

## 7. Limitations of the study

This study is a retrospective chart review including a small number of patients in the CS-AKI group. Also the incidence and risk factors of CS-AKI changes with the definition used. The incidence of AKI in our study is probably lower than if we had used the risk, injury, failure, loss, and end-stage kidney disease (RIFLE) criteria. This may have affected our risk factor association as well.

## Acknowledgments

The authors would like to thank Mr. John Cyrus who provided us editorial assistance.

## Authors’ contribution

All the authors have contributed towards performing the study and preparation of the manuscript and they all have approved the latest version of the article.

## Conflicts of interest

The authors declared no competing interests.

## Ethical considerations

Ethical issues (including plagiarism, data fabrication, double publication) have been completely observed by the authors.

## Funding/Support

This study had no external source of funding.
